# An Inherited Heteroplasmic Mutation in Mitochondrial Gene COI in a Patient with Prostate Cancer Alters Reactive Oxygen, Reactive Nitrogen and Proliferation

**DOI:** 10.1155/2013/239257

**Published:** 2012-12-27

**Authors:** Rebecca S. Arnold, Qian Sun, Carrie Q. Sun, Jendai C. Richards, Sean O'Hearn, Adeboye O. Osunkoya, Douglas C. Wallace, John A. Petros

**Affiliations:** ^1^Department of Urology, School of Medicine, Emory University, 1365 Clifton Rd. Building B, Atlanta, GA 30322, USA; ^2^Winship Cancer Institute, Emory University, Atlanta, GA 30322, USA; ^3^Center for Molecular and Mitochondrial Medicine and Genetics (MAMMAG), University of California Irvine, Irvine, CA 92697, USA; ^4^Department of Pathology and Laboratory Medicine, Emory University, Atlanta, GA 30322, USA; ^5^Department of Urology, The Atlanta VA Medical Center, Decatur, GA 30033, USA; ^6^Center for Mitochondrial and Epigenomic Medicine, Children's Hospital of Philadelphia and Department of Pathology and Laboratory Medicine, University of Pennsylvania, Philadelphia, PA 19104, USA; ^7^Department of Pathology, University of Pennsylvania, Philadelphia, PA 19104, USA

## Abstract

Mitochondrial DNA (mtDNA) mutations have been found in many cancers but the physiological derangements caused by such mutations have remained elusive. Prostate cancer is associated with both inherited and somatic mutations in the cytochrome c oxidase (COI) gene. We present a prostate cancer patient-derived rare heteroplasmic mutation of this gene, part of mitochondrial respiratory complex IV. Functional studies indicate that this mutation leads to the simultaneous decrease in cytochrome oxidation, increase in reactive oxygen, and increased reactive nitrogen. These data suggest that mitochondrial DNA mutations resulting in increased reactive oxygen and reactive nitrogen generation may be involved in prostate cancer biology.

## 1. Introduction

The mitochondrion contains the only functional DNA outside the eukaryotic cell nucleus and mutations in this genome have been linked to a vast array of human disease including pediatric neurologic disease, degenerative muscular disease, and blindness in the pediatric population and more recently to chronic diseases of adults including diabetes, Alzheimer's dementia, Parkinson's disease, cardiovascular disease, and cancer [1]. Because of the critical role the mitochondria play in normal human cell physiology, severe mutations that cause dramatic derangements of mitochondrial function can be fatal [2]. Indeed, recent evidence indicates that severe mutations are culled from the germ line through depletion of mutant germ cells in the ovary [3]. Severe mutations can exist and even be passed between generations if they exist in a state of heteroplasmy where some copies of the genome contain the mutation and some are wild type. For this reason, one indication that a mitochondrial DNA (mtDNA) mutation is potentially pathogenic is that it exists in a heteroplasmic state, though this is not strictly necessary in all cases.

Prostate cancer is one example of an adult disease tightly linked to ageing for which there is evidence linking both inherited and somatic mtDNA mutations to disease [4]. Mutations in the mitochondrial cytochrome c oxidase subunit I (COI) gene are found at disproportionately high rates in prostate cancer patients compared to either the general population or rigorously defined controls without prostate cancer [5]. A population-based case-control study of African Americans found 2 single nucleotide polymorphisms in this gene significantly associated with prostate cancer (*P* < 0.05) and in strong linkage disequilibrium with each other (*r* > 0.6) [6]. MtDNA mutations are easily detected in early stage prostate cancer tissues and can also be found in the serum and urine from the same individuals [7]. MtDNA content has been linked to androgen responsiveness of prostate cancer cells *in vitro* [8] and analysis of clinical samples suggests that mtDNA content is higher in prostate cancer compared to adjacent normal prostatic tissue in the same individual [9]. A specific mtDNA deletion mutation may have utility in identifying adult cancer not apparent in clinical prostate biopsies [10]. 

While multiple independent investigators have confirmed mtDNA mutations in prostate cancer, there is little understanding of the cell biologic and biochemical consequences of specific prostate cancer-associated mtDNA mutations. We investigated the mtDNA from a patient with prostate cancer and found a heteroplasmic missense mutation in the mitochondrial COI gene that impairs the oxidation of cytochrome c (respiratory complex IV inhibition) and increases the generation of both reactive oxygen species (ROS) and reactive nitrogen species (RNS).

## 2. Materials and Methods

### 2.1. Cytochrome c Oxidase Subunit I (COI) Gene Sequencing

The mtDNA region encompassing COI was amplified using a forward primer starting at nucleotide position (np) 5772 (5′ AGG TTT GAA GCT GCT TCT TC 3′) and a reverse primer ending at np 7600 (5′ CGC TGC ATG TGC CAT TAA GA 3′). The template was denatured at 95°C for 7 min and primers extended for 35 cycles of 94°C for 1 min, then 55°C for 1 min, and 72°C for 1 min. Both strands of the COI polymerase chain reaction (PCR) product were cycle-sequenced using the slip primers in the forward direction starting at np 6080 (5′ TCT ACA ACG TTA TCG TCA CA 3′) and at np 6930 (5′ TGC AGT GCT CTG AGC CCT AG 3′) and in the reverse direction starting at np 6340 (5′ CTA GGT GTA AGG AGA AGA TG 3′) and at np 7150 (5′ GAT TTA CGC CGA TGA ATA TG 3′). The templates were denatured at 96°C and primers extended in the presence of “Big Dye Terminators” for 25 cycles of 96°C for 10 sec, then 55°C for 5 sec, and 60°C for 4 min. The reactions were chilled to 4°C, and the excess dye terminators removed by Centri-Sep Columns. The trace files were determined using an Applied Biosystems (ABI) Prism 3100 genetic analyzer, analyzed using Sequencher gene analysis software v 4.1 (Gene Codes, Ann Arbor, MI), and interpreted within the context of MITOMAP (http://www.mitomap.org/). Full mtDNA sequencing was performed as described above using primer sets that span the full length of the mitochondrial DNA [11].

### 2.2. Laser Capture Microdissection (LCM)

LCM was carried out by the Winship Cancer Institute Pathology Core using the Molecular Machines & Industries, Inc. Cell cut laser microdissector to isolate pure populations of prostatic epithelium (malignant and benign) and stromal cells. DNA was purified from LCM of frozen tissue using PicoPure DNA Extraction Kit (Arcturus, Mt View, CA) according to the recommended protocol.

### 2.3. Epstein-Barr Transformation

 Lymphocytes were isolated from whole blood by centrifugation and diluted with phosphate buffered saline (PBS). Red cells were lysed by the addition of H_2_O. After 20 s, osmolarity was restored with 10x concentrated PIPES (piperazine-*N*,*N*′-bis-[2-ethano-sulfonic-acid]) buffer, centrifuged, and the pellet was resuspended in RPMI with fetal bovine serum (FBS) and incubated for 45 min in 5% CO_2_ at 37°C. The nonadherent cells were washed and collected and lymphocytes pelleted. Lymphocytes were infected with the B95-8 strain of Epstein-Barr Virus (EBV) and cultured in RPMI-1640 medium supplemented with 10% heat-inactivated fetal bovine serum, 2 mM l-glutamine, 100 U/mL penicillin, and 100 mg/mL streptomycin, at 37°C in a humid atmosphere saturated with 5% CO_2_ until cryopreservation or use [12].

### 2.4. Mitochondrial Preparation and Isolation

Mitochondria were isolated by cell fractionation and centrifugation. All procedures were carried out on ice. Cells were pelleted and washed in cold PBS and resuspended in 10 volumes of isolation buffer (250 mM sucrose, 10 mM HEPES, 1 mM EDTA, pH 7.35). Cell lysis was performed with 5 passes of a hand held Dounce homogenizer and centrifuged at 500 g (4°C) for 5 min. The supernatant was reserved and the pellet was resuspended in isolation buffer, rehomogenized, and centrifuged. This process was repeated a third time and supernatants combined and centrifuged 12,000 g at 4°C for 10 min to pellet mitochondria which were then washed in isolation buffer and repelleted three times. Following the final wash, mitochondria were resuspended in reaction buffer (1 mL/mL of original cell pellet), and frozen until assayed.

### 2.5. Cytochrome c Oxidase Activity

Cytochrome c oxidase activity of isolated mitochondrial preparations was measured by dual wavelength spectrophotometry (550/540 nm) as previously described [13]. Briefly, mitochondria were suspended in reaction buffer (250 mM Sucrose, 10 mM HEPES, 1 mM MgCl_2_, pH 7.35). An aliquot of mitochondria suspension (~300 ug mitochondrial protein) was added to the cuvette containing reaction buffer plus ~5 uM reduced cytochrome c in a final volume of 700 uL. The oxidation of cytochrome c was recorded over time and the cytochrome c oxidase specific activity was calculated. Mitochondrial protein was determined by the Bradford method [14].

### 2.6. Cybrid Formation

Mitochondrial donors (patient lymphoblasts) were enucleated by short term incubation with Actinomycin D, which was subsequently removed from the culture medium by centrifugation and washing. These cytoplasts were then rescued by polyethylene glycol (PEG)-induced fusion with 143B rho zero cells. Fusions are monitored by phase contrast microscopy and isolated by ring cloning. Clones were expanded and genotyped to assure that the donor mtDNA had been incorporated (sequencing) and that a single nucleus was present (Karyotyping).

### 2.7. Flow Cytometric Assay for ROS and RNS

Peroxides Assay: Lymphoblasts were grown in suspension in flasks. When cells were in growth phase, 1 × 10^6^ cells per sample were removed, pelleted, and resuspended in 2 *μ*M CM-DCFDA (5-(and -6)-chloromethyl-2′,7′-dichlorodihydrofluorescein diacetate) (Life Technologies, Grand Island, NY). Cells were placed in a dark room at room temperature while gently rocking for 30 minutes (CM-DCFDA). Following this incubation, samples were kept on ice until counting on a Becton Dickinson FACScan flow cytometer. 10,000 cells were counted and analyzed by FlowJo 7.6.4 to compare mean values of DCF fluorescence intensity. All samples were repeated in triplicate. Some sample tubes also contained 10 M FCCP (Mesoxalonitrile 4-trifluoromethoxyphenylhydrazone) and were preincubated with the lymphoblasts prior to the addition of CM-DCFDA. Cybrid cell lines were also treated with CM-DCFDA. Briefly, adherent cells were grown to 70% confluency, washed with PBS, and cells were removed from the plate by the addition of trypsin-EDTA (Mediatech, Inc, Manassas, VA). Cells were pelleted at 400 g for 5 minutes at room temperature and the cells were resuspended in CM-DCFDA and assay continued as described above. Mitochondrial Superoxide Assay: Media (RPMI 1640 containing 10% fetal bovine serum) was replaced on cells in growth phase with media containing 5 *μ*M MitoSOX (Life Technologies). Cells were incubated in the dark at 37°C, 5% CO_2_ for 10 minutes. Following this incubation, cells were harvested by trypsin digestion, pelleted, and resuspended in HANKS Buffer containing 10% FBS. Samples were kept on ice until counting on a Becton Dickinson LSRII flow cytometer as described above. NO Assay: Media (RPMI 1640 containing 10% fetal bovine serum) was replaced on cells in growth phase with media containing 4 *μ*M 4-amino-5-methylamino-2′,7′-difluorofluorescein diacetate (DAF-FM DA) (Life Technologies). Cells were incubated in the dark at 37°C, 5% CO_2_ for 30 minutes, DAF-FM DA was removed and replaced with media and incubated in the dark at 37°C, 5% CO_2_ for a additional 20 minutes. Following this incubation, cells were harvested by trypsin digestion, pelleted, and resuspended in HANKS Buffer containing 10% FBS. Samples were kept on ice until counting on a Becton Dickinson LSRII flow cytometer as described above. Time between dye loading and reading was 30 minutes and all samples were treated identically. The DAF experiment was done in the presence of serum.

### 2.8. Cell Proliferation Assay

Cell were plated in 6 well plates, 18,000 cells per well in triplicate for each time point and grown in RPMI 1640 (Mediatech) containing 15% Fetal Bovine Serum. Cells were harvested after 1, 2, 3, or 4 days and cell number was determined using FluoReporter Blue Fluorometric dsDNA Quantitation Kit (Life Technologies) according to manufacturer's protocol.

### 2.9. Reverse Transcription and PCR

 In order to determine which isoform of Nitric Oxide Synthase (NOS) is present in our cybrid cell lines we performed RT-RCR on RNA from the cybrid cell lies and a positive control. cDNA was obtained from the RNA of 6124WT, 6124Mut, and BT474 (positive control) by reverse transcription using Advantage RT for PCR from Clontech (a Takara Bio Company, Mountain View, CA) according to protocol. The presence of NOS1, NOS2, and NOS3 were examined. Using the Amplitaq Gold Kit (Life Technologies), reactions were as follows: cDNA, 1XBuffer, 1.5 mM MgCl_2_, 0.2 mM each dNTPs, 150 nM each forward and reverse primers for NOS1 (Forward 5′ CGA CAC CAC TAG CAC TTA CCA G 3′; Reverse 5′ CAG ACT CGG AAG TCG TGC TTG 3′), NOS2 (Forward 5′ TCG GCT GCA GAA TCC TTC ATG A 3′; Reverse 5′ CAT TGT CTT GCG CAT CAG CAT AC 3′) or NOS3 (Forward 5′ GAA GCA CCT GGA GAA TGA GCA G 3′; Reverse 5′ CTT CAC TCG CTT CGC CAT CAC 3′) and 5 units of Taq for a final volume of 100 uL. Primers were designed to cross two introns and amplify all major isoforms of NOS1. The PCR reaction included an initial cycle of 95°C for 10 min followed by 40 cycles of 95°C for 30 sec and 61°C for 30 sec, 72°C for 1 min. A 3% agarose gel was run after PCR. The presence of NOS1, NOS2, and NOS3 were indicated by the presence of a 261 nt band, 288 nt or a 274 nt band, respectively.

### 2.10. Western Blot Analysis

 Whole-cell extracts were obtained by lysing cells with lysis buffer containing 50 mmol/L, Tris Base, 5 mmol/L EGTA, 150 mmol/L NaCl, and 1% Triton X-100 (pH 7.4). One tablet of protease inhibitor (Roche Applied Science, Indianapolis, IN) was dissolved in 7 mL of lysis buffer. Total protein 30 *μ*g/well was loaded in 4–12% gradient NuPAGE MES SDS gel (Life Technologies) and transferred into Immun-Blot PVDF Membrane (BIO-RAD, Hercules, CA). The membrane was immunoblotted with anti-PARP (Cell Signaling Technology, Danvers, MA) at 1 : 2000 dilution and anti-*β*-actin (Sigma, St. Louis, MO) at 1 : 2000 dilution. Immunodetection was completed by using the corresponding secondary horseradish peroxide- conjugated antibodies (Amersham, Piscataway, NJ). Horseradish peroxide activity was detected using enhanced chemiluminescence from ECL Western Blotting Analysis System (Amersham).

### 2.11. Mice

 Male nu/nu mice, 6–8-weeks old, were purchased from Charles River Laboratories (Wilmington, MA) and housed in ventilated cages under sterile conditions. For surgical manipulation, mice were anesthetized with an intramuscular injection of a mixture of ketamine hydrochloride, Xylazine, and Acepromazine.

### 2.12. *In Vivo* Tumor Study

 Mice were injected subcutaneously in the neck with 3 separate 6124WT clones for a total of 29 mice and 3 separate 6124Mut clones for a total of 30 mice. 2.5 × 10^5^ cells, resuspended in PBS, were injected per mouse. Mice were checked daily for tumor growth. When tumors were visually observed they were measured twice a week and the tumor volumes calculated using the formula [length × (width)^2^]/2 [15]. Experiment was repeated 2 additional times. Mice were sacrificed when the tumors reached 10% of body weight. Tumor tissues were dissected for further study.

## 3. Results

### 3.1. Mutation Analysis

**Figure 1:**
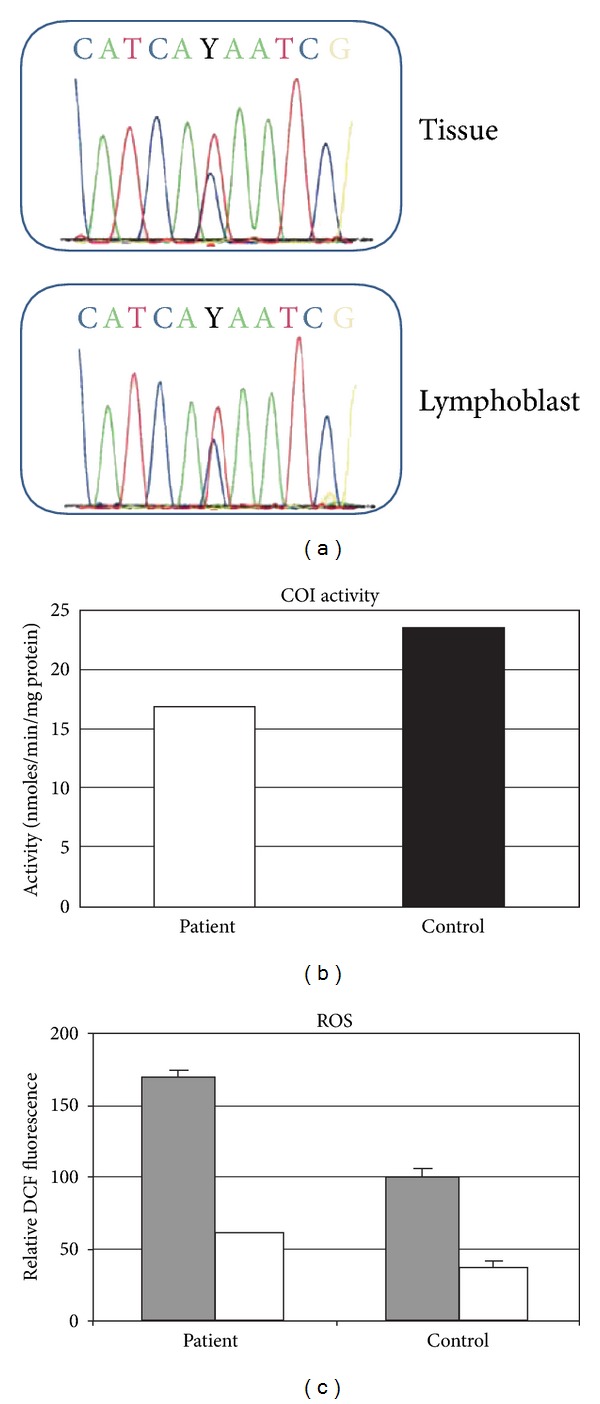
Detection of heteroplasmic point mutation of mitochondrial cytochrome oxidase subunit I (COI) mitochondrial gene from a single individual with prostate cancer. (a) Sequencing chromatograms of prostatic tissue and an Epstein-Barr transformed lymphoblast cell line show approximately equal levels of both the wild type (T) and mutant (C) DNA base. (b) Activity of cytochrome oxidase measured in isolated mitochondria prepared from the patient's heteroplasmic lymphoblasts (see Section 2 for details) compared to the average of two-lymphoblast lines from controls with only the wild type base at position 6124. (c) Flow cytometric analysis of DCF fluorescence in the patient's heteroplasmic lymphoblasts compared to the average of two-lymphoblast lines from controls with only the wild type base at position 6124 (gray bars). Cells were also analyzed for DCF fluorescence in the presence of FCCP (white bars). Error bars represent the standard deviation of 2–4 data points.

A 60-year old patient underwent radical prostatectomy after presenting to his primary care physician with an elevated-serum-prostate-specific antigen (PSA = 5.5 ng/mL) that prompted a prostate biopsy revealing a Gleason 6 prostatic adenocarcinoma. Histopathologic examination of the radical prostatectomy specimen revealed a Gleason's score 6 (moderately differentiated-grade G2) conventional (acinar) prostatic adenocarcinoma involving ~5% of the left lobe of the prostate with negative surgical margins (pathologic stage pT2a). Follow-up of over 7 years with history, physical examinations and serum PSA determinations found the patient to be without evidence of prostate cancer recurrence. There was no family history of prostate cancer and the patient was otherwise healthy. DNA from the prostate tissue was the first specimen to be sequenced from this patient and this demonstrated a heteroplasmic point mutation at nucleotide position (np) 6124 of the mitochondrial genome. The sequencing chromatogram demonstrated approximately equal proportions of the wild type base (T) and mutant base (C) (Figure 1(a)). The mutation changes amino acid 74 from the nonpolar, methionine (sulfur side chain) to the polar, threonine (hydroxyl side chain). This is a highly conserved amino acid, being methionine in 95% of all species (*N* = 61) for which the sequence has been determined. This conservation index (CI) of 95 is at the very upper end of what is observed for “adaptive mutations” (CI = 85 ± 9%) and significantly above the CI of “neutral polymorphisms” (CI = 23 ± 15%), making it highly likely to be functional in terms of mitochondrial physiology [1]. Another indicator of the uniqueness of this base and amino acid change is that this patient remains the only example of this base change in MITOMAP and mutation at this nucleotide position has not been found in the 2704 complete mtDNA sequences reported in the online database mtDB [16], where 2704 out of 2704 individuals have the wild type base (T). Subsequent sequencing of patient peripheral blood DNA, lymphoblast cell lines, and laser capture microdissection of various cellular compartments of the prostate all revealed similar levels of heteroplasmic mutation at this base, confirming inheritance in the germ line and maintenance of the mutation in the prostate. The entire mitochondrial DNA sequence for this patient was determined and all changes from the revised Cambridge reference sequence are shown in Table 1. The mutation at n.p. 6124 was the only heteroplasmic mutation and the only mutation that had not been previously found in the 2407 complete mtDNA sequences published in the online database mtDB.

### 3.2. Cytochrome Oxidase Enzyme Activity

In order to study the biochemical consequences of this mutation we studied reactive oxygen production and enzymatic activity of respiratory complex (RC) IV (cytochrome oxidoreductase). The COI polypeptide forms the catalytic core of this enzyme. Cytochrome oxidation was measured in mitochondria isolated from the patient's lymphoblast cell line and compared to other lymphoblast cell lines from two unrelated, individuals' lymphoblast lines, sequence proven to be wild type at the COI locus (Figure 1(b)). The mutation is associated with a 29% decrease in COI activity.

### 3.3. Reactive Oxygen

Mutations in the COI polypeptide that lead to decreased RCIV activity could potentially lead to increased reactive oxygen [17]. In order to determine if this was occurring, we assayed the lymphoblast cell line of the patient for increased peroxide levels using dichlorofluorescin (DCF) and compared the results to the two-wild type patients described above. There was a marked increase (1.75 fold) in DCF fluorescence in the lymphoblast cell line containing the mutation compared to wildtype (Figure 1(c), gray bars). The majority of the increase in DCF fluorescence can be attributed to the mitochondria as demonstrated when oxidative phosphorylation is inhibited with FCCP (white bars), DCF fluorescence levels are decreased to similar levels. More detailed analysis of the effect of the 6124 mutation on ROS generation was obtained in the cybrid experiments, results highlighted below (“Cybrid ROS and RNS”).

### 3.4. Cybrid Generation

 In order to eliminate the potential confounding effects of the nuclear genome we made cytoplasmic hybrids (cybrids) that combined either pure mutant or pure wild type genomes from this patient with a stable nuclear background (143 B cells). The resultant pair of cybrids thus have the exact same nucleus (different from the patient), and the exact same mitochondrial DNA sequence except for the single base mutation at n.p. 6124. MtDNA genotypes were sequence verified in cybrids.

### 3.5. Cybrid *In Vitro* Proliferation

**Figure 2:**
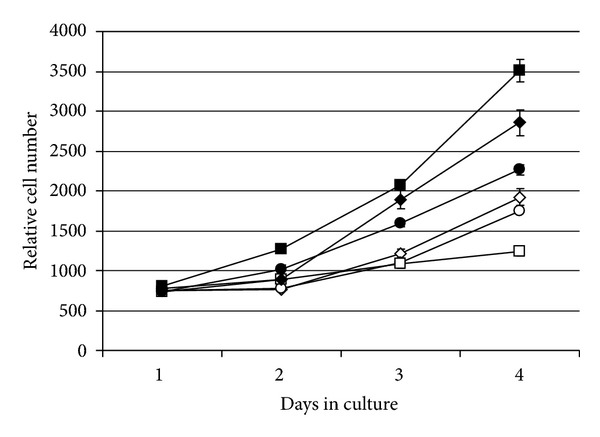
Cybrid cell lines with the T6124C mutation show increased proliferation. Proliferation was measured in 3 separate 6124WT clones and 3 separate 6124Mut clones using FluoReporter Blue Fluorometric dsDNA Quantitiation Kit (see Section 2). Symbols represent the following clones: *◊*-6124WT3; ◯-6124WT5; □-6124WT6, ◆-6124Mut1; *⚫*-6124Mut2; ■-6124Mut4. Error bars represent the standard error of the mean of triplicate data.

Three cybrids 6124 wild type clones and three 6124 mutant clones were analyzed for proliferation in culture. All three mutant 6124 cybrid clones grew faster than the three wild type clones with an average doubling time of 1.57 ± 0.12 days compared to 2.81 ± 0.56 days, mutant to wild type, respectively, (*P* ≤ 0.098) (Figure 2).

### 3.6. Cybrid ROS and RNS

**Figure 3:**
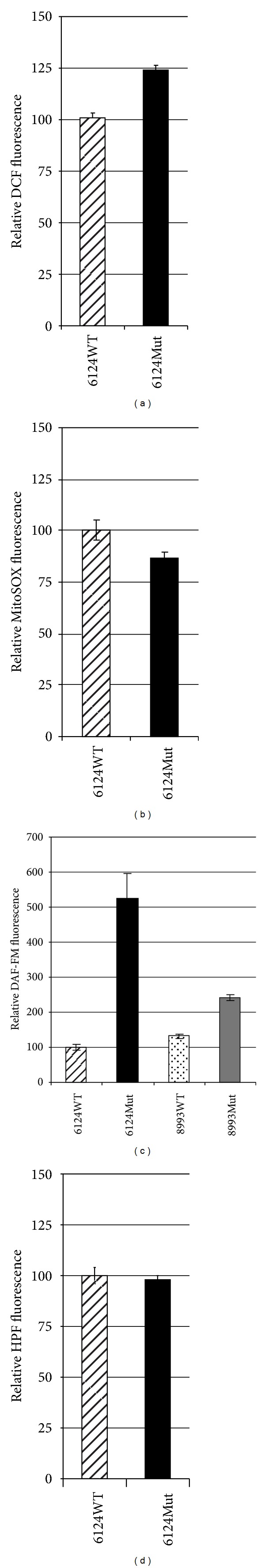
Peroxide and Nitric Oxide are elevated in 6124 mutant cybrid cells. (a) Peroxide levels are elevated in 6124Mut cell lines as measured by flow cytometric analysis of DCF fluorescence. 143B cybrids cell lines containing either the wild type base at position 6124WT or the mutation at position 6124Mut were analyzed. The average DCF fluorescence of five wild type and six mutant clonal cell lines are shown. Mutant cybrids produce significantly more peroxides (*P* ≤ 0.0001). Error bars represent the standard error of the mean of 10 data points (WT) and 12 data points (Mut). (b) Mitochondrial superoxide levels are decreased in 6124Mut cell lines compared to 6124WT (*P* ≤ 0.04). The average MitoSOX fluorescence of three wild type and three mutant clonal cell lines are shown. Error bars represent the standard error of the mean of 9 data points each WT and Mut. (c) NO levels are highly elevated in 6124Mut cells compared to 6124WT as measured by DAF-FM Fluorescence (*P* ≤ 0.00001). For comparison, 143B cybrids cell lines containing a separate patient mtDNA with either a mutation at position 8993 (8993Mut) or wild type 8993 (8993WT) is shown. Error bars represent the standard error of the mean of 9 data points each 6124WT, 6124Mut, and 3 data points each 8993WT and 8993Mut. (d) Hydroxyl radicals and peroxynitrite anions as measured by hydroxyphenylfluorescein (HPF) remain unchanged. Error bars represent the standard error of the mean of 9 data points each 6124WT and 6124Mut.

We then compared multiple wild type and mutant clones for reactive oxygen species (ROS) and reactive nitrogen species generation using flow cytometry and ROS and RNS sensitive fluorescent dyes. In order to study the cellular peroxide levels in cells that were either entirely mutant or entirely wild type at this base, cybrid cell lines were made and multiple clones assayed by DCF fluorescence. Overall, 6 individual mutant clones showed a consistent increase in peroxide levels when compared to 5 individual wildtype clones (data not shown). When averaging the relative level of fluorescence in all wildtype clones compared to mutant clones, the mutation is associated with a statistically significant (*P* ≤ 0.0001) increase in DCF fluorescence (Figure 3(a)). Similarly, in order to determine mitochondrial superoxide levels, there was a slight overall decrease in mitochondrial superoxide in the 6124Mut cell lines when compared to 6124WT (*P* ≤ 0.04) (Figure 3(b)). A substantial and significant increase was observed in nitric oxide (NO) levels in the same 6124Mut and 6124WT cell lines (Figure 3(c)). When treated with DAF-FM diacetate, 6124Mut cell lines showed an average of 5.2-fold increase in NO levels compared to 6124WT (*P* ≤ 0.00001). For comparison, NO was also measured cybrids containing 8993WT or Mut. 8993Mut cells had 1.8-fold higher NO when compared to 8993WT (*P* ≤ 0.0003). Finally, there were no differences in the overall levels of hydroxyl radicals and peroxynitrite anions as measured by hydroxyphenylfluorescein (HPF) (Figure 3(d)).

### 3.7. Nitric Oxide Synthases (NOS) in Cybrids Cell Lines

**Figure 4:**
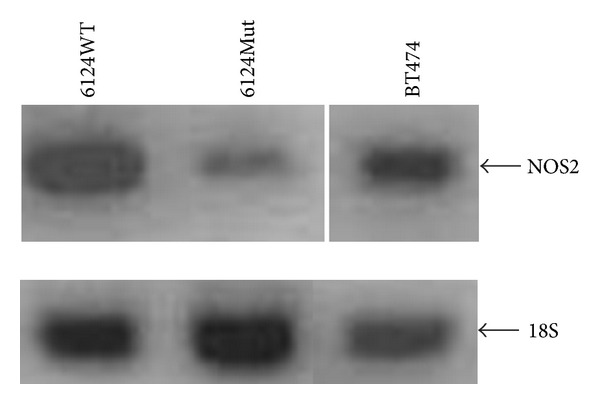
iNOS RNA is present in the cybrids cells. Reverse transcription followed by standard PCR was performed with iNOS specific primers. iNOS RNA was present in both 6124WT and 6124Mut cell lines. The breast cancer cell line BT474 is used as a robustly positive control for iNOS RNA. and 18S RNA was used as a quality control.

In order to determine the possible source of the increased NO, we harvested RNA from the 6124WT and 6124Mut cybrids cell lines, followed by reverse transcription and PCR. There were no detectable levels of NOS1 or NOS3 observed (data not shown). However, NOS2 was demonstrated to be in both WT and Mut cell lines (Figure 4). The breast cancer cell line BT474 is used as a robustly positive control for NOS2 RNA.

### 3.8. Apoptosis

**Figure 5:**
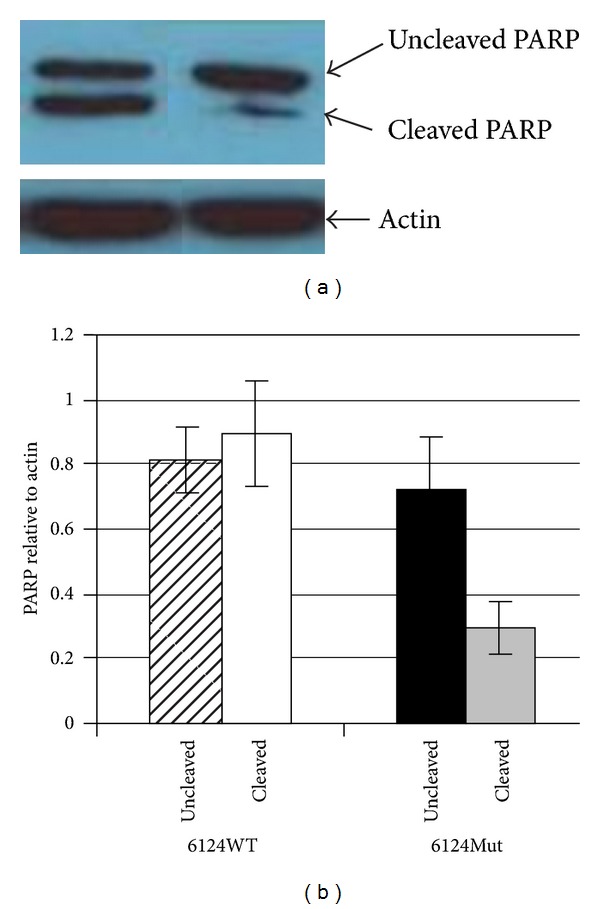
PARP cleavage is decreased in 6124Mut cybrids cells. (a) Western Blot analysis of uncleaved and cleaved PARP in 6124WT (left) and 6124Mut (right) cells. Figure is representative of three 6124WT and three 6124Mut clones. (b) Densitometric analysis of Western Blot results of three 6124WT and three 6124Mut clones using ImageJ software. Error bars represent the standard error of the mean of the three WT and three Mut clones.

PAPR (poly ADP ribose polymerase) cleavage was analyzed to determine if changes in cellular proliferation were in part due to changes in apoptosis. Western blot analysis of PARP cleavage demonstrates that PARP cleavage was reduced in 6124Mut cybrids compared to 6124WT (Figure 5).

### 3.9. *In Vivo* Proliferation

**Figure 6:**
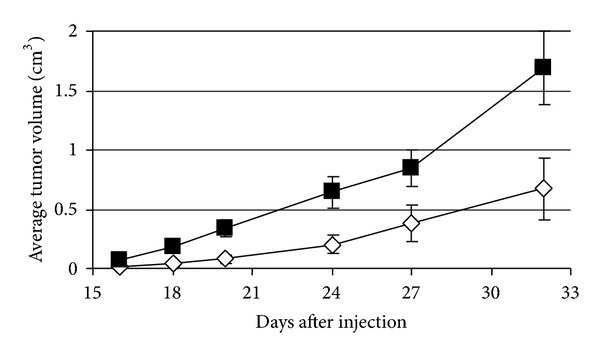
6124Mut cell lines grow faster in nude mice. Growth curves of tumor xenografts in nude mice. Each line represents a cohort of 29-30 animals injected with the 6124WT cybrids cells (*◊*) or 6124Mut cybrids cells (■). Error bars denote the standard error of the mean for each cohort at each time. Data are representative of 3 individual experiments.

 6124Mut cybrid cell lines grew faster *in vivo* when compared to 6124WT cybrids cells (Figure 6). There was some variability within each group as to the number and size of tumor, but at all time points there was a statistically significant increase in the size of tumors from 6124Mut cybrids cell lines (*P* ≤ 0.001 at day 32). The animals injected with cybrids showed no systemic symptoms in response to tumor injection or growth. 

## 4. Discussion

The mitochondrially encoded COI gene was first implicated in cancer biology in 1998 when a somatically acquired chain termination mutation was reported in colon cancer by the Vogelstein group at Johns Hopkins [18]. In 2005 an analysis of 260 patients with prostate cancer revealed that 31 (12%) had an inherited mutation in COI compared to <2% of no-cancer controls. Similarly, a case-control analysis of African-American men revealed that two COI gene single nucleotide polymorphisms (SNPs) (T6221C and T7389C) were significantly associated with prostate cancer (*P* < 0.05) and in strong linkage disequilibrium with each other (*r* ^2^ > 0.6). It is therefore likely that COI gene mutations predispose individuals to the development of prostate cancer. For this reason we have sequenced the COI gene from 482 prostate cancer patients and identified missense mutations in 116 (24.1%) [19]. The patient reported in this paper is part of that cohort. 

The biochemical analysis of mtDNA mutations requires that viable cells are obtained from patients with such mutations and that potentially confounding nuclear events are controlled. This is made possible by the combination of capturing clinically relevant mutations in patient lymphoblast cell lines and the subsequent formation of cybrids with a common nuclear background. The cybrid formation process has the further advantage of allowing the mutant base to be studied separately from the wild type base. This paper documents that a prostate cancer-associated COI mutation affects the normal functioning of respiratory complex (RC) IV (cytochrome oxidase) in at least two distinct ways: decreasing the rate of mitochondrial cytochrome c oxidation and increasing the rate of ROS and NO generation in intact cells. It therefore seems likely that both of these effects are a direct consequence of the mutation. As Figure 3 depicts, not all ROS are affected equally by the 6124 mutation. In particular, peroxides are elevated, superoxide is depressed and there is no difference in hydroxyl radicals. One possible explanation for the decrease in superoxide levels in the presence of abundant superoxide dismutase that results in the rapid conversion of superoxide to hydrogen peroxide. Hydrogen peroxide is stable and able to accumulate while superoxide anion is transient and highly reactive.

The possible tumorigenic effects of increases in ROS are well known and include (at least) an increased rate of DNA mutations and ROS-induced promitogenic signaling [20–22]. These are common findings in prostate cancer and other solid tumors [23, 24]. It is likely that the cumulative effect over a lifetime of a tonic increase in cellular ROS increases the chances of malignant transformation. It is uncertain whether the prostate is more susceptible to this influence than other organs, but the association of COI mutations with disease suggests some specificity.

The possible mechanism by which a decreased efficiency of oxidative phosphorylation is related to malignant transformation is less obvious. It is probably relevant to the so called “Warburg Effect” wherein tumor cells exhibit defective oxidative phosphorylation and increased glycolysis as a primary means of ATP generation [25]. It is possible that inherited or somatically acquired mtDNA mutations are partially or wholly responsible for this effect. It is possible that the decrease in oxidative phosphorylation is not in and of itself causally related to the increased risk of cancer, but that the relation to cancer risk is conferred predominantly by the increased ROS generation and that compromises to oxidative phosphorylation are merely a bystander effect of no direct consequence to tumorigenesis. This remains to be determined.

The possible tumorigenic effects of increases in RNS are also well known. Nitric oxide (NO) is generated enzymatically by synthases (NOS), which oxidize L-arginine to L-citrulline. The inducible form, iNOS, is present in a variety of cell types. Over the past 20 years iNOS expression has been associated with various human tumors including: breast, brain, lung, prostate, colorectal, and melanoma [26–32]. NO action is concentration-dependent. Increased cGMP-mediated ERK phosphorylation is associated with low levels of NO in cancer cells whereas HIF-1a stabilization is associated with intermediate levels [33]. Though NO has been shown to induce apoptosis it can transcriptionally enhance MMP-1 via the ERK and p38 MAPK pathways resulting in tumor progression and also can result in the overproduction of VEGF [33]. Although NO levels are transient, iNOS generated NO fluctuations varying from seconds to days can influence the antiapoptotic/proapoptotic effects of NO [33].

We have demonstrated that the T6124C (M74T) mutation was inherited in a heteroplasmic state by a patient that developed prostate cancer and that the mutation not only causes increased ROS and nitric oxide but also induces increased cellular proliferation, decreased apoptosis, and increased *in vivo* tumor growth. These are quintessential characteristics of the malignant phenotype. These results are concordant with other studies that demonstrate a profound effect of mtDNA mutations on tumor cell biology. In 2008 Ishikawa demonstrated that mtDNA mutations determined the metastatic potential of lung cancer cell lines independent of the characteristics of the cancer cell nucleus. Specifically, high-metastatic cell lines lost their metastatic potential when the mtDNA was replaced with mtDNA from low-metastatic cell lines and low-metastatic cell lines acquired high-metastatic capabilities when mtDNA from high metastatic lines were inserted [34]. The metastatic capacity could be eliminated by treatment with antioxidants indicating the central importance of ROS-induced signaling in metastasis. Similarly, an ATP6 (ATP synthase subunit 6) missense mutation in the mitochondrial genome (T8993G) causes increased ROS in prostate cancer cell lines and increased tumor growth [5].

## 5. Conclusions

Mitochondrial DNA from a prostate cancer patient with an inherited-heteroplasmic-mtDNA mutation in COI, the catalytic core of mitochondrial respiratory complex IV was studied. In the laboratory, this mutation was found to simultaneously decrease the activity of the respiratory complex as measured by the rate of cytochrome c oxidation and to increase the rate of mitochondrial reactive oxygen generation. Other mutation-induced biochemical changes included increased generation of nitric oxide. Cells harboring the mutation proliferated faster *in vitro* and caused increased tumor growth *in vivo*. These findings suggest a possible molecular substrate (mtDNA mutation) for the “Warburg effect” of anaerobic metabolism exhibited by some tumors and increased cellular reactive oxygen, a common finding in solid tumors.

## Figures and Tables

**Table 1:** Patient mitochondrial DNA mutations. Patient peripheral blood, lymphoblast, and cybrid mtDNA was sequenced in its entirety. All changes from rCRS are shown as is the region in which the change occurs and the amino acid alteration when applicable. AI (allelic index) is the measure of the frequency of the mutation when compared to mtDB—Human Mitochondrial Genome Database [16]. Cybrid mtDNA was identical to peripheral blood with the exception of n.p. 6124 at which point the cybrids were determined to be either homoplasmic mutant or wild type.

ΔrCRS	Amino acid	Region	AI
A73G	noncoding	D-loop	83.4
A263G	noncoding	D-loop	99.7
309insC	noncoding	D-loop	
311insC	noncoding	D-loop	
523delA	noncoding	D-loop	
524delC	noncoding	D-loop	
G709A	noncoding	12S rRNA	16.4
A750G	noncoding	12S rRNA	99.2
A1438G	noncoding	12S rRNA	96.9
G1888A	noncoding	16s rRNA	5.3
A2706G	noncoding	16s rRNA	80.5
T4216C	Tyr → His	ND1	9.0
A4769G	Met	ND2	99.0
A4917G	Asn → Asp	ND2	4.8

T6124 T&C	Met → Thr	COI	unique

C7028T	Ala	COI	81.3
8270-8278del	noncoding	NC7	
G8697A	Met	ATPase6	4.7
G8854A	Ala → Thr	ATPase6	0.1
A8860G	Thr → Ala	ATPase6	99.8
T10463C	noncoding	tRNA Arg	4.7
A11251G	Leu	ND4	8.7
G11719A	Gly	ND4	77.7
A11812G	Leu	ND4	3.3
G13368A	Gly	ND5	4.9
A14233G	Asp	ND6	3.4
C14766T	Ile → Thr	Cytb	77.4
G14905A	Met	Cytb	5.1
A15326G	Thr → Ala	Cytb	99.4
C15452A	Leu → Ile	Cytb	8.7
A15607G	Lys	Cytb	5.5
G15928A	noncoding	tRNA Thr	4.9
T16126C	noncoding	D-loop	8.9
T16189C	noncoding	D-loop	28.0
C16278T	noncoding	D-loop	7.7
C16294T	noncoding	D-loop	5.7
C16296T	noncoding	D-loop	2.4
T16519C	noncoding	D-loop	59.7
